# Metabolic flux in macrophages in obesity and type-2 diabetes

**DOI:** 10.3389/jpps.2024.13210

**Published:** 2024-06-26

**Authors:** Angela Wong, Qiuyu Sun, Ismail Ibrahim Latif, Qutuba G. Karwi

**Affiliations:** ^1^ Department of Pediatrics, Faculty of Medicine and Dentistry, University of Alberta, Edmonton, AB, Canada; ^2^ Department of Microbiology, College of Medicine, University of Diyala, Baqubaa, Diyala, Iraq; ^3^ Division of BioMedical Sciences, Faculty of Medicine, Memorial University of Newfoundland, Saint John’s, NL, Canada; ^4^ Department of Pharmacology, College of Medicine, University of Diyala, Baqubaa, Diyala, Iraq

**Keywords:** macrophage, metabolism, obesity, type-2 diabetes, glucose, fatty acids, ketones, amino acids

## Abstract

Recent literature extensively investigates the crucial role of energy metabolism in determining the inflammatory response and polarization status of macrophages. This rapidly expanding area of research highlights the importance of understanding the link between energy metabolism and macrophage function. The metabolic pathways in macrophages are intricate and interdependent, and they can affect the polarization of macrophages. Previous studies suggested that glucose flux through cytosolic glycolysis is necessary to trigger pro-inflammatory phenotypes of macrophages, and fatty acid oxidation is crucial to support anti-inflammatory responses. However, recent studies demonstrated that this understanding is oversimplified and that the metabolic control of macrophage polarization is highly complex and not fully understood yet. How the metabolic flux through different metabolic pathways (glycolysis, glucose oxidation, fatty acid oxidation, ketone oxidation, and amino acid oxidation) is altered by obesity- and type 2 diabetes (T2D)-associated insulin resistance is also not fully defined. This mini-review focuses on the impact of insulin resistance in obesity and T2D on the metabolic flux through the main metabolic pathways in macrophages, which might be linked to changes in their inflammatory responses. We closely evaluated the experimental studies and methodologies used in the published research and highlighted priority research areas for future investigations.

## Introduction

Obesity and type 2 diabetes (T2D) are conditions marked by insulin resistance and persistent low-grade inflammation [1–3]. Chronic tissue inflammation is now recognized as an essential characteristic of obesity and T2D, affecting insulin-target tissues such as adipose tissue, liver, muscle, and heart. The recruitment, accumulation, and activation of pro-inflammatory macrophages in metabolic tissues play important roles in driving this chronic low-grade inflammation. Although other types of immune cells also contribute to these inflammatory processes, macrophages are primary effector cells known to be closely associated with the development of cardiometabolic disease, including obesity and T2D (see for [3–7] review). Macrophages are crucial immune cells involved in immune response [8] and play important roles in tissue repair and maintaining the body’s homeostasis [9]. The inflammatory responses of macrophages are supported by different metabolic pathways (glycolysis, glucose oxidation, fatty acid oxidation, ketone oxidation, and amino acid oxidation) that adjust to their polarization state [10]. Importantly, the metabolic flux through different metabolic pathways could be influenced by the tissue microenvironment, the availability of oxidative substrates, and neurohormonal status. In addition, altered macrophage energy metabolism can impact tissue repair, inflammatory responses, and the severity of insulin resistance [2, 11–13].

Earlier studies suggested that pro-inflammatory (also called M1-like or classical) macrophages are highly glycolytic, while anti-inflammatory (also called M2-like or alternative) macrophages are highly oxidative [14–16]. However, this classification has been challenged as simplistic, as emerging evidence shows that macrophages have a very complex and dynamic metabolic profile that influences their activity. Importantly, “immunometabolism” has emerged as a prerequisite trigger of macrophage activation and phenotype. Nevertheless, minimal research has been steered to understand how the metabolic phenotype of macrophages influences disease progression [17]. Therefore, a better understanding of macrophage metabolism may shed an innovative light on the pathological basis of disease and lead to the future development of macrophage-targeted treatment approaches. In this mini-review, we discussed how carbon flux through the main metabolic pathways in macrophages is perturbed in obesity and T2D and how that influences the inflammatory response, activity and metabolic profile of macrophages.

## Glycolysis

**FIGURE 1:**
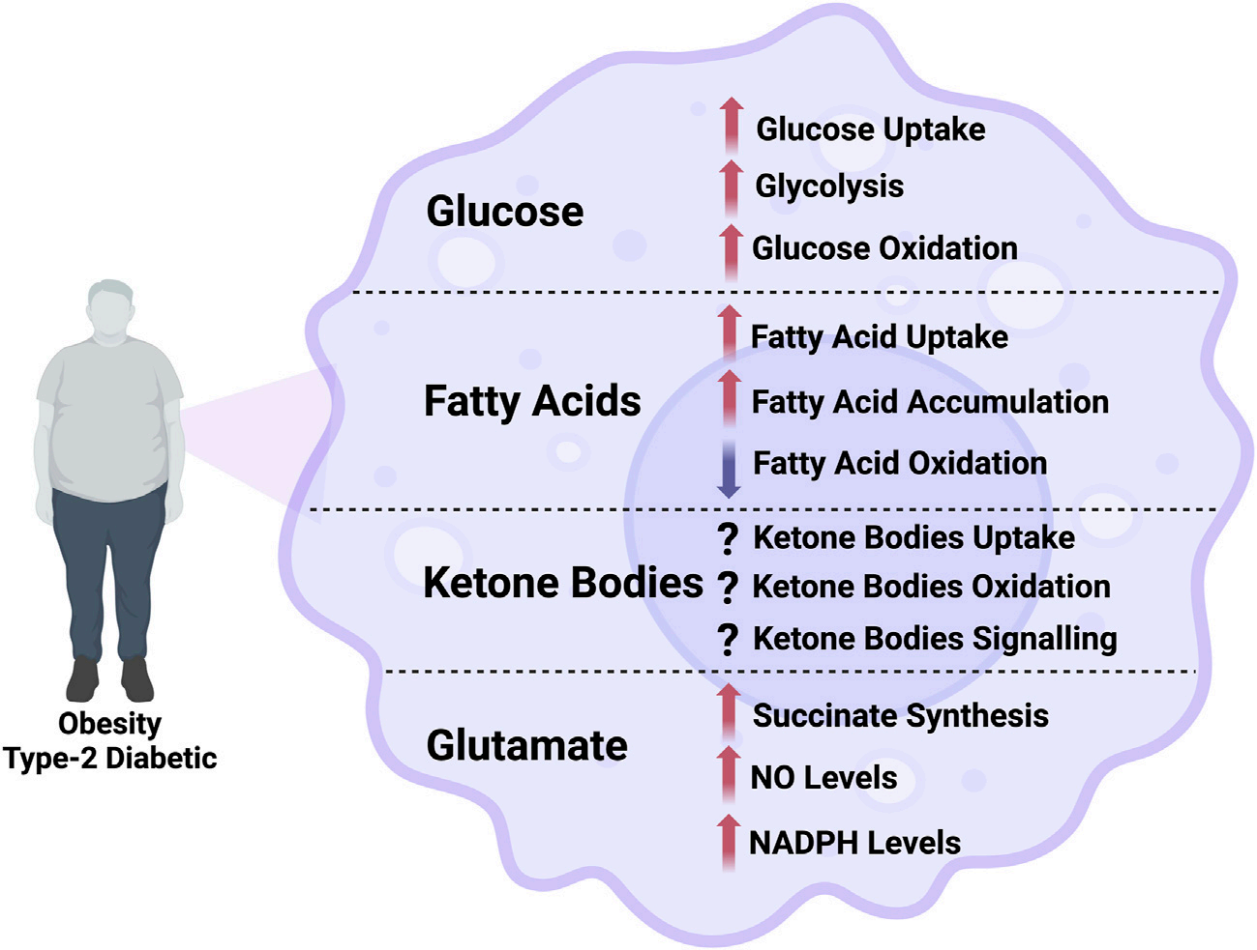
Illustration of the alterations in the flux through different metabolic pathways in macrophage in obesity and type-2 diabetes. NO, nitric oxide; NADPH, nicotinamide adenine dinucleotide phosphate.

Insulin signalling is a key modulator of macrophage metabolism by regulating glucose uptake and oxidation [18, 19]. However, in obesity and T2D, the insulin signalling pathway is impaired, leading to insulin resistance in macrophages [18, 19]. Interestingly, this results in the upregulation of glycolysis and glucose uptake, which is associated with proinflammatory macrophage polarization [20]. This metabolic switch is crucial for the host defence mechanisms of macrophages, such as cytokine production and phagocytosis. Increased glucose influx in insulin-resistant macrophages is facilitated by upregulated glucose transporter 1 (GLUT1) expression [21, 22]. In high-fat diet (HFD) fed mice, the overexpression of GLUT1 in the pro-inflammatory macrophages also led to a hyperinflammatory state with the elevated secretion of inflammatory mediators and increased reactive oxygen species (ROS) production [23]. The metabolomic analysis also demonstrated an increased glucose uptake in the GLUT1 overexpressed macrophages enhances glucose flux through the pentose phosphate pathway, where glucose is utilized to generate NADPH for use in biosynthetic pathways and ROS production [23]. Upon activation, proinflammatory macrophages undergo a “respiratory burst,” also called “glycolytic burst,” driven by augmented NADPH oxidase activity to generate large amounts of ROS as a defence mechanism against pathogens [24]. To support redox balance, the glycolysis-PPP axis is triggered in response to M1 polarization, presumably to support the increased generation of NADPH for use by NADPH oxidase as well as glutathione production used by macrophages to protect from the excessive amounts of superoxide being produced [24]. This suggests that GLUT1-mediated glucose metabolism plays an important part in driving the pro-inflammatory state of macrophages in obesity and T2D (Figure 1). Consistent with that, genetic deletion of GLUT1 in bone marrow-derived macrophages (BMDMs) displays marked reductions in the classically activated pro-inflammatory markers and associated oxidative stress [25]. Inhibiting glycolysis or treating macrophages with an antioxidant (N-acetyl-cysteine) reversed GLUT1-mediated pro-inflammatory elevations [23].

Increased glucose availability in T2D-induced hyperglycemic conditions can also promote the formation of advanced glycation end products (AGEs) in macrophages [26, 27]. AGEs are pathogenic factors that trigger the activation of a number of signalling pathways in macrophages, including the NF- κB and the MAPK signalling pathways under hyperglycemia conditions [28, 29]. Enhanced AGEs in BMDMs increase interleukin 6 (IL-6) and tumour necrosis factor-alpha (TNF-α) production [30]. AGEs also enhance the polarization of macrophages toward the pro-inflammatory state by inducing the expression of pro-inflammatory molecules in T2D [29, 31]. Hypoxia-inducible factor 1 subunit alpha (HIF1α) also plays a critical role in increasing glycolytic flux and abrogating oxidative metabolism (OXPHOS) in macrophages in obesity and T2D [32, 33]. To further support this, HIF-1α gene deletion in mice protected against HFD-induced adipose tissue inflammation and systemic insulin resistance [34].

## Glucose oxidation

Increasing glucose uptake in macrophages by overexpressing GLUT1 enhances glucose flux through cytosolic glycolysis and mitochondrial glucose oxidation [23]. While these metabolic changes are associated with promoting the pro-inflammatory phenotype of macrophages, it is still unclear whether the increase in glycolysis and/or glucose oxidation are essential for promoting pro-inflammatory macrophages. Min et al. demonstrated that pyruvate dehydrogenase kinase (PDK), which inhibits the pyruvate dehydrogenase-mediated conversion of cytosolic pyruvate to mitochondrial acetyl-CoA, functions as a metabolic checkpoint in inflammatory macrophages [35]. Deletion of PDK2 and PDK4 completely abolishes the development of pro-inflammatory macrophages in HFD-induced insulin resistance [35]. Inhibition of macrophage glucose oxidation is also associated with weight loss, reduced insulin resistance, and decreased adipose tissue inflammation [35]. Taken together, inhibiting macrophage glucose oxidation is a potential target to limit the severity of insulin resistance in obesity and T2D (Figure 1).

It has been suggested that the neurogenic locus notch homolog protein 1 (NOTCH1) signalling pathway contributes to the activation of mitochondrial glucose oxidation in obesity. For instance, Xu et al. showed an enhanced macrophage glucose oxidation in obesity that is mediated, at least in part, by increased recruitment of the NOTCH1 intracellular domain (NICD1) to nuclear and mitochondrial genes that encode respiratory chain components [36]. This effect also involved NOTCH-dependent induction of pyruvate dehydrogenase phosphatase 1 (Pdp1) expression, pyruvate dehydrogenase activity, and glucose flux to the tricarboxylic acid (TCA) cycle [36]. This enhancement of glucose oxidation is associated with augmented levels of mitochondrial DNA transcription in pro-inflammatory macrophages, thus causing enhanced mitochondrial ROS levels [36]. Therefore, glucose oxidation may be a target in macrophages to alleviate insulin resistance and inflammation induced by HFD.

## Fatty acid oxidation

Obesity and T2D-induced lipid accumulation in adipose tissue are associated with elevated fatty acid uptake, increased macrophage infiltration, and decreased fatty acid oxidation [37, 38]. Moreover, increased lipolysis at the adipose tissue level has been linked to lipid-droplet accumulation in adipose tissue macrophages (ATMs) and obesity-induced inflammation [39, 40]. Studies have shown that macrophages develop a distinct phenotype in obesity exemplified by increased lysosomal acid type lipase, fatty acid receptor, ATP-binding cassette A1 expressions, and inflammatory cytokines (IL-1β and TNF-α) [41, 42]. In addition to acting as fuel for activated macrophages, excessive lipid intake is also shown to be a primary factor that causes pro-inflammatory macrophage polarization in obesity and T2D [43].

The fatty acid translocase CD36 binds to fatty acids and is important for fatty acid uptake at the myocardium, skeletal muscle, gastrointestinal tract, liver, and adipose tissue level [44–46]. In macrophages, CD36 primarily acts as a scavenger receptor, recognizing specific self and nonself molecular patterns and triggering internalization and inflammatory signalling pathways to eliminate pathogens and altered self components, such as apoptotic cells [47]. CD36 cooperates with toll-like receptor (TLR)-4 and −6 to trigger inflammatory responses to altered self-components oxidized LDL (ox-LDL) and amyloid-β [48]. CD36 also acts as a coreceptor with TLR2 and −6 in recognizing microbial diacylglycerides [49]. The deletion of CD36 in BMDMs displayed improved insulin signalling and reduced macrophage infiltration in adipose tissue [50, 51]. This may be attributed to the potential role of upregulated fatty acid uptake in mediating obesity-induced inflammation and insulin resistance (Figure 1), although this is yet to be directly investigated. Recent studies have shown that CD36 is important in the mitochondrial metabolic switch from oxidative phosphorylation to superoxide production in response to ox-LDL and that mitochondrial reactive oxygen species positively correlate with macrophage CD36 expression [52].

Fatty acid oxidation occurs within the mitochondria, and several steps are required to activate the fatty acids and transport them into the mitochondria [53–55]. Cytosolic fatty acids are first esterified to fatty acyl-CoA (a process consuming two high-energy phosphate bonds as ATP is converted to AMP), followed by the transfer of the fatty acid moiety to carnitine via the action of carnitine palmitoyl-transferase 1 (CPT1) to form fatty acylcarnitine. CPT1, residing on the outer mitochondrial membrane, works collaboratively with carnitine acyltranslocase and carnitine palmitoyl-transferase 2 (CPT2), residing on the inner mitochondrial member, to transfer fatty acylcarnitine into the mitochondrial matrix, where it is converted back to fatty acyl-CoA. These acyl-CoAs then undergo β-oxidation to produce reduced equivalents (NADH and FADH_2_) for the electron transport chain (ETC), as well as acetyl-CoA for the tricarboxylic acid (TCA) cycle. Malonyl-CoA can be generated by acetyl-CoA carboxylase (ACC) from cytosolic acetyl-CoA [56, 57]. Malonyl-CoA can be converted back to acetyl-CoA by malonyl-CoA decarboxylase (MCD) [58, 59]. While the role of ACC enzymes (ACC1 and ACC2) in macrophages is not fully defined, individual deletion of ACC1 or ACC2 in the myeloid lineage is a prerequisite for the function of highly proliferative T cells, but not for macrophages [60]. Recent studies have shown that ACC is required for the early metabolic switch to glycolysis and remodelling of the fatty acid metabolism in macrophages. Using mice with myeloid-specific deletion of both ACC isoforms, ACC deficiency impairs macrophage innate immune functions, including bacterial clearance [61]. Myeloid-specific deletion or pharmacological inhibition of ACC in mice attenuated LPS-induced expression of proinflammatory cytokines interleukin-6 (IL-6) and IL-1β. In contrast, pharmacological inhibition of ACC increased susceptibility to bacterial peritonitis in wild-type mice [61].

It has been shown that the overabundant influx of fatty acids largely shifts from fatty acid oxidation to triglyceride, phospholipid, and ceramide synthesis, contributing to macrophage lipotoxicity [62–64]. This also contributes to macrophage insulin resistance and the consequential promotion of mitochondrial dysfunction in macrophages [63]. While indirect evidence suggests that altered fatty acid metabolism influences macrophage activation in obesity and T2D [65], it is unknown whether macrophage fatty acid oxidation is upregulated or downregulated in obesity and T2D. Malandrino et al. reported that enhancing fatty acid oxidation in human ATMs reduces ROS, endoplasmic reticulum stress, and pro-inflammatory responses of macrophages [37]. In line with that, the deletion of macrophage carnitine palmitoyl transferase 1A (CPT1A) catalyzes the transfer of the long-chain acyl group in acyl-CoA ester to carnitine. This allows fatty acids to enter the mitochondrial matrix for oxidation and exacerbates the accumulation of diacylglycerols and triacylglycerols after palmitate treatment *in vitro* [66]. CPT1A deletion also increased pro-inflammatory signalling, cytokine expression and endoplasmic reticulum stress after palmitate treatment [66]. Consistent with that, decreasing triglyceride and free cholesterol levels in macrophages mitigates the activation of pro-inflammatory macrophages, supporting the link between lipid accumulation in these cells and the switch to the pro-inflammatory polarization state (Figure 1) [43]. The intermediates of the biosynthetic pathways for triacylglycerol or phospholipids can affect the inflammatory and insulin signalling pathways in different tissues. Saturated fatty acids are also precursors of sphingolipids; in particular, ceramides are strongly linked to insulin resistance and inflammation. TLR4 signalling can trigger ceramide biosynthesis, promoting insulin resistance by activating protein phosphatase 2A and protein kinase C-zeta, ultimately inhibiting Akt [67]. Ceramides may also activate the inflammasome, inducing IL-1β secretion in macrophages, which can blunt insulin signalling [68]. These results might suggest that enhancing macrophage fatty acid oxidation could reduce macrophage activation and mitigate insulin resistance in obesity and T2D. However, this needs to be directly investigated in future research.

Insulin-resistant adipocytes also release greater levels of fatty acids while activating ATMs, leading to an intensified cycle of inflammation through the indirect stimulation of the macrophage toll-like receptor 4 (TLR4) [69, 70]. This causes the initiation of the Jun N-terminal kinase and inhibitor of κB kinase (JNK/IKK- κB) pathways followed by inflammatory cascades [71]. Suganami et al. showed that coculturing obese mice-derived hypertrophied adipocytes and macrophages augments the production of TNF-α in macrophages [69]. The released TNF- α in turn promotes the secretion of FFAs and inflammatory changes in adipocytes [69]. To further support this, TLR4-deletion in BMDMs inhibits saturated FA-induced inflammation via inhibiting palmitate-induced activation of the JNK signalling pathway [72]. Therefore, this shift in fatty acid metabolism towards greater production of inflammatory lipids and levels of FFAs in ATMs exacerbate insulin resistance in obesity and T2D [73, 74]. Glucose-6-phosphate dehydrogenase (G6PD) is a key enzyme that produces cellular NADPH, which is required for cellular redox potential and the biosynthesis of fatty acids and cholesterol. Macrophage G6PD levels are increased in the adipose tissue of obese animals, and G6PD mRNA levels positively correlated with those of pro-inflammatory genes [75]. Lipopolysaccharide (LPS) and free fatty acids, which initiate pro-inflammatory signals, stimulated macrophage G6PD. Overexpression of macrophage G6PD potentiated the expression of pro-inflammatory and pro-oxidative genes responsible for the aggravation of insulin sensitivity in adipocytes. Macrophage G6PD stimulates the p38 mitogen-activated protein kinase (MAPK) and NF-κB pathways, causing a vicious cycle of oxidative stress and pro-inflammatory cascade [75].

Macrophage infiltration to the myocardium also increases due to obesity and T2D-induced inflammation. Saturated fatty acids contribute to myocardial inflammation by inducing the release of inflammatory molecules through pattern recognition receptors (PRRs) in macrophages [71, 72, 76]. Enhanced fatty acid delivery to the heart in obesity and T2D causes uncoupling of fatty acid oxidation from adenosine diphosphate phosphorylation and promotes further mitochondrial dysfunction in cardiomyocytes [77–80]. The imbalance in cardiac lipid metabolism leads to the accumulation of ceramides and diacylglycerols in the hearts of obese and diabetic patients [77, 81–83]. Ceramides activate the NLRP3 inflammasome and further promote cardiac lipotoxicity in palmitate-exposed human cardiac cells and HFD-fed mice [84]. The interplay between altered cardiac fatty acid oxidation and macrophage infiltration into the myocardium in obesity and T2D is an interesting scope for future investigations.

## Ketone oxidation

Ketone bodies are organic compounds mainly produced by the liver by breaking down fatty acid molecules. The three major ketone bodies are β-hydroxybutyrate (βOHB), acetoacetate (AcAc), and acetone. βOHB is first oxidized to AcAc by βOHB dehydrogenase, followed by conversion to acetoacetyl-CoA by succinyl-CoA:3 oxoacid-CoA transferase (SCOT). The end-product of ketone oxidation is acetyl-CoA, which has a similar fate as acetyl-CoA produced from fatty acid or glucose oxidation. While there is limited data regarding how ketone metabolism is regulated in macrophages and how it might be altered in obesity and T2D, a recent study using isotope tracking LC/MS untargeted metabolomics showed that macrophages could oxidize ketones with preferential utilization of AcAc compared to βOHB [85]. Preclinical studies have shown that enhancing circulating AcAc levels ameliorates diet-induced hepatic fibrosis, and this protective effect is abolished in macrophage-specific SCOT knock-out mice [85]. These findings suggest that increasing macrophage ketone oxidation plays a critical role in modifying the inflammatory responses of macrophages [85].

Furthermore, another study demonstrated that administration of βOHB increases the expression of IL-10 and arginase 1, markers of the inflammation-resolving state of macrophages and the resolution of damaged intestinal tissue in a mouse model of inflammatory bowel disease [86]. In addition to supporting macrophage energetics, βOHB could also influence macrophage activity by acting as a signalling molecule (see for [87] review). For instance, the inhibitory effect of βOHB on the NLRP3 inflammasome in BMDMs is mediating by acting as a ligand of macrophage GPR109A, a member of the hydrocarboxylic acid GPR sub-family expressed in adipose tissues (white and brown) and immune cells [88]. While these findings suggest that ketone bodies elicit a predominantly anti-inflammatory response, augmented circulating ketone levels in diabetic patients may trigger a pro-inflammatory response (Figure 1) [89–91]. Future studies are required to delineate whether this modulatory effect of ketones on macrophage function is mediated by enhancing increased macrophage ketone oxidation and/or via acting as a signalling molecule [87]. It would also be important to determine how modulating macrophage ketone oxidation affects macrophage function in obesity and T2D. Taken together, this encouraging emerging evidence suggests that ketone bodies have an inhibitory effect on the inflammatory response of macrophages, which might be beneficial against the low-grade chronic inflammation in obesity and T2D and its detrimental effects. However, it is yet to be determined whether ketone bodies modulate macrophage responses by serving as oxidative substrates to modulate macrophage ATP production or acting as signalling molecules.

## Amino acid oxidation

The flux through different amino acid metabolic pathways changes according to macrophage phenotype. For instance, arginine is converted to nitric oxide (NO) via inducible NO synthase (iNOS) in pro-inflammatory M1-like macrophages [92, 93]. In contrast, it is converted to proline and polyamines via arginase-1 in inflammation-resolving M2-like macrophages (Figure 1) [94]. Glutamine is the most abundant amino acid in the body and acts as a main source of carbon and nitrogen for cells. While serum glutamine levels are lower in patients with obesity and diabetes [95], studies have demonstrated that glutamine metabolism is altered in macrophages on exposure to M1-or M2-polarizing agents. For instance, glutamine is channelled into the TCA cycle for synthesizing succinate in M1-like macrophages, leading to a marked intracellular accumulation of succinate, enhancing proinflammatory cytokine production [33]. On the contrary, glutamine is critical for acquiring the M2 polarization state. It is mostly converted to α-ketoglutarate in M2-like macrophages and enhances the production of key anti-inflammatory cytokines through its role in protein glycosylation (Figure 1) [96, 97]. Consistent with that, glutamine deprivation impairs the expression of M2-like macrophage markers *in vitro* [97]. Macrophages collected from obese insulin-resistant Zucker rats had a significantly lower NO production than those collected from lean control rats [98]. Interestingly, incubating macrophages from obese insulin-resistant Zucker rats with glutamine increases NO production [98]. Since NO is produced using arginine as a precursor, these findings suggest crosstalk between arginine and glutamine metabolism in macrophages, highlighting the importance of differential use of amino acids to modulate macrophage responses in insulin resistance conditions. It is important to mention that the action of iNOS in converting arginine to NO depends on the availability of an important cofactor, NADPH. Macrophages can utilize glucose and glutamine to synthesize NADPH [99]. Macrophages collected from insulin-resistant rats have impaired NO production due to impaired synthesis of NADPH and less activation of iNOS in the absence of glutamine [98]. Therefore, suggesting that glutamate contributes to NADPH synthesis in insulin-resistant macrophages seems plausible.

Augmented levels of branched-chain amino acids (BCAAs), namely leucine, isoleucine, and valine, and their respective metabolites, namely branched-chain keto acids (BCKAs), have been linked with metabolic alterations, insulin resistance, and a predisposition to T2D [100]. It has been shown that BCAAs play a role in modulating inflammatory responses in immune cells. However, the data regarding whether high levels of BCAAs promote pro-inflammatory or anti-inflammatory immune cells are inconclusive. For instance, enhancing BCAA levels promotes oxidative stress, inflammation and human peripheral blood mononuclear cell migration [101]. In contrast, high levels of BCAA exert anti-inflammatory and anti-genotoxic activity in LPS-stimulated macrophages [102]. Whether these effects are mediated via enhancing BCAAs and BCKAs contributions to mitochondrial oxidative metabolism as fuel or via acting as signalling molecules in macrophages remains to be determined in future investigations.

## Discussion

Recent studies have linked macrophage metabolic processes to their inflammatory behaviour. They demonstrated that macrophages could switch from promoting tissue protection to contributing to disease development by altering the flux through different metabolic pathways. Although macrophage insulin signalling is impaired in obesity and T2D, insulin-resistant macrophages have increased glucose uptake, glycolysis, and glucose oxidation. Macrophage fatty acid uptake is also increased, but it seems uncoupled to fatty acid oxidation, which is decreased in obesity and T2D. Instead, fatty acids are converted to triacylglycerol and ceramide accumulation, which contribute to lipotoxicity in insulin-resistant macrophages. Arginine and glutamate metabolism also have divergent effects in pro- and anti-inflammatory macrophages in obesity and T2D. Understanding the complex metabolic profile of different macrophage phenotypes will help characterize how different oxidative substrates could influence macrophage responses. In addition, understanding the metabolic reprogramming behind macrophage responses will help identify new avenues for therapeutic intervention to combat obesity and T2D.
