# Corrigendum: Metabolic flux in macrophages in obesity and type-2 diabetes

**DOI:** 10.3389/jpps.2025.15426

**Published:** 2025-09-03

**Authors:** Angela Wong, Qiuyu Sun, Ismail Ibrahim Latif, Qutuba G. Karwi

**Affiliations:** ^1^ Department of Pediatrics, Faculty of Medicine and Dentistry, University of Alberta, Edmonton, AB, Canada; ^2^ Department of Microbiology, College of Medicine, University of Diyala, Baqubaa, Diyala, Iraq; ^3^ Division of BioMedical Sciences, Faculty of Medicine, Memorial University of Newfoundland, Saint John's, NL, Canada; ^4^ Department of Pharmacology, College of Medicine, University of Diyala, Baqubaa, Diyala, Iraq

**Keywords:** macrophage, metabolism, obesity, type-2 diabetes, glucose, fatty acids, ketones, amino acids

In the published article, there was an error regarding the **Affiliations** for Qutuba G. Karwi. As well as having **Affiliation 3**, they should also have “Department of Pharmacology, College of Medicine, University of Diyala, Baqubaa, Diyala, Iraq.”

The authors apologize for this error and state that this does not change the scientific conclusions of the article in any way. The original article has been updated.

